# Supplementary material for: Novel Time-Lapse Parameters Correlate with Embryo Ploidy and Suggest an Improvement in Non-Invasive Embryo Selection
Source: J Clin Med. 2023 Apr 19;12(8):2983. doi: 10.3390/jcm12082983 (PMC10146271; doi:10.3390/jcm12082983)
Supplement: Supplementary file 1 [file jcm-12-02983-s001.zip › jcm-2310309-supplementary.pdf]

Table S1. Nomenclature of morphokinetic dynamic events.

t_0	Time of IVF or mid-point of micro/injection (ICSI)
t_{PB2}	The second polar body is completely detached from the oolemma
t_{PN}	Fertilization status is confirmed by the appearance of first PN
t_{PNf}	The time when both (and the last) PNs are no longer visible
t_n	First time frame at which an embryo reaches n number of blastomeres
st_2	First evidence of cytoplasmatic movements prior to first cytokinesis (Video S1)
t_{SC}	First evidence of compaction
t_M	Time of completion of compaction process *
t_{SB}	Initiation of blastulation (first frame in which the blastocoel is visible)
t_B	Full blastocyst (blastocoele cavity fills the embryo and the inner cell mass and trophectoderm tissues are distinguishable from each other)
cc1 (t_2–t_{PNf})	Duration of first cell cycle
cc2 (t_3–t_2)	Duration of second cell cycle
cc3 (t_5–t_3)	Duration of the third cell cycle
t_5–t_2	Interval between 2 and 5 cells (which combines the concepts of cell cycle and synchrony)
s2 (t_4–t_3)	Synchronization of cell divisions (synchronicity of the two blastomere divisions within the second cell cycle)
s3 (t_8–t_5)	Synchronization of cleavage pattern (the synchronicity of the four blastomere divisions within the third cell cycle)
t_B–t_{SB}	Duration of blastulation

Note: Table adapted from [8]. Some of the times may change from one study to another depending on the t_0 used and the normalization applied. These inconsistencies must be taken into account when using time-lapse-derived models and algorithms. * Times regarding morula formation may be difficult to use as strict marking times as cells may continue fusing and being excluded even after the start of cavitation, which hardens the annotation of the real t_M time. Therefore, other authors and we consider it difficult to use t_M in algorithms or models when predicting euploidy or other outcomes [8].

Table S2. Baseline characteristics of patients involved in the study.

		**Euploid**	**Aneuploid**	***p*-Value**
No. of patients	75			
No. of PGT cycles	85			
Female age (years) (95% CI)	38.0 (37.6–38.4)	37.8 (37.3–38.3)	38.8 (38.4–39.2)	0.002
Fertilization technique				
IVF (cycles; embryos)	49; 202	77 (38.1%)	125 (61.9%)	ns
ICSI (cycles; embryos)	36; 172	81 (47.1%)	91 (52.9%)	
Day of biopsy				
D5	299	129 (81.6%)	170 (18.4%)	ns
D6	75	29 (78.7%)	46 (21.3%)	
Indication				
AMA (cycles (%))	53 (62.4%)			
RPL (cycles (%))	7 (8.2%)			
AMA + RPL (cycles (%))	11 (12.9%)			
RIF (cycles (%))	3 (3.5%)			
Altered karyotype (cycles (%))	10 (11.8%)			
Elective (cycles (%))	1 (1.2%)			
No. of embryos analyzed (mean per cycle; SD)	374 (4.4; 2.6)			
Mean number of euploid embryos per cycle	1.8 ± 1.8			
Euploidy rate per cycle (95% CI)	38.3% (31.4–45.0)			
Aneuploidy rate per cycle (95% CI)	61.7% (55.0–68.5)			

Note: PGT: preimplantation genetic testing; IVF: in vitro fertilization; ICSI: intracytoplasmic sperm injection; AMA: advanced maternal age; RPL: recurrent pregnancy loss; RIF: recurrent implantation failure; ns: not significant.
