# Supplementary material for: Novel Time-Lapse Parameters Correlate with Embryo Ploidy and Suggest an Improvement in Non-Invasive Embryo Selection
Source: J Clin Med. 2023 Apr 19;12(8):2983. doi: 10.3390/jcm12082983 (PMC10146271; doi:10.3390/jcm12082983)
Supplement: Supplementary file 1 [file jcm-12-02983-s001.zip › Video captions.pdf]

Video S1. *Start of t_2 (st_2).* Video shows the first cytoplasmic movements prior to first cytokinesis. Hours indicated in the video (top left) refer to hours post-insemination without normalization. St_2 and t_2 (red and blue, respectively) are indicated with the specific time appearing at the right side of the embryo when the phenomena are taking place.

Video S2*. Pattern 0 embryo examples.* Example of two embryos classified in pattern 0 group. No halo disappearance prior to the first cell cleavage is detected; cells are divided maintaining the cytoplasmic halo.

Video S3. *Pattern 1 embryo examples.* Representative videos of three embryos displaying pattern 1 st_2. A: Halo disappearance is visible simultaneously to PN breakout, followed by smooth cell cleavage. B: embryo presents halo disappearance followed by non-patterned cytoplasmic movements. C: Chaotic cytoplasmic waves can be observed before direct unequal cleavage.

Video S4. *Pattern 2 embryo examples.* Representative time-lapse videos of embryos classified as pattern 2. A: Clockwise circular wave observed prior to first cell division, 3 complete rounds observed. B: Embryo presents membrane smooth blebbing prior to counterclockwise circling movements (2 turns observed), followed by cell division.
